# The effectiveness of two energy drinks on selected indices of maximal cardiorespiratory fitness and blood lactate levels in male athletes

**Published:** 2010

**Authors:** Nader Rahnama, Abbas Ali Gaeini, Fahimeh Kazemi

**Affiliations:** a Associate Professor, Faculty of Physical Education and Sport Sciences, University of Isfahan, Isfahan, Iran; b Professor, Faculty of Physical Education and Sport Sciences, University of Tehran, Tehran, Iran; c MS, Faculty of Physical Education and Sport Sciences, University of Tehran, Tehran, Iran

**Keywords:** Energy Drink, Caffeine, Taurine, VO_2max_, Blood Lactate, Male Athletes

## Abstract

**BACKGROUND:**

Consumption of energy drinks has become widespread among athletes. The effectiveness of Red Bull and Hype energy drinks on selected indices of maximal cardiorespiratory fitness and blood lactate levels in male athletes was examined in this study.

**METHODS:**

Ten male student athletes (age: 22.4 ± 2.1 years, height: 180.8 ± 7.7 cm, weight: 74.2 ± 8.5 kg) performed three randomized maximal oxygen consumption tests on a treadmill. Each test was separated by four days and participants were asked to ingest Red Bull, Hype or placebo drinks 40 minutes before the exercise bout. The VO _2max_, time to exhaustion, heart rate and lactate were measured to determine if the caffeine-based beverages influence performance. ANOVA test was used for analyzing data.

**RESULTS:**

A greater value was observed in VO _2max_and time to exhaustion for the Red Bull and Hype trial compared to the placebo trial (p < 0.05). No significant difference was found in pre-and post-test heart rate for two drinks (p > 0.05). For blood lactate levels no significant changes were observed before and two minute after the test (p > 0.05).

**CONCLUSIONS:**

Ingestion of Red Bull and Hype prior to exercise testing is effective on some indices of cardiorespiratory fitness but not on the blood lactate levels.

For some years the beverages denominating “energy drinks” have become widespread both in recreational and trained athletes, because of their proposed ergogenic effects. A variety of energy drinks are currently available on the market today and are publicized to increase the energy level of the individuals consuming it. Sport drinks, such as Gatorade and Powerade, have been designed to refuel athletes during and after their performance. Specifically, they are designed to have optimal levels of carbohydrate for glycogen replenishment, electrolytes for ion maintenance, and prevent dehydration. Contrary, energy drinks are reported to increase mental alertness and physical performance during exercise. This is because energy drink manufacturers add ergogenic ingredients such as carbohydrates, caffeine, taurine and vitamins. These ingredients have been proposed to interact with each other to provide a stimulant effect.^1^ Furthermore, the manufacturers of these drinks claim a variety of benefits in their advertisements and although energy drinks have been sold worldwide for more than a decade, there is a paucity of evidence supporting their effectiveness of enhancing aerobic performance in athletes.^2^

Geiß et al (1994)^3^ and Alford et al (2001)^4^ have shown beneficial effects of Red Bull on performance. Geiß et al (1994)^3^ reported improved endurance time and lower heart rates on Red Bull which was attributed to increased catecholamine circulation while, Alford et al (2001)^4^ indicated improved aerobic, anaerobic and psychomotor performance. Taken together, these results suggest that Red Bull has the potential to improve physical and mental performance.^4^ Baum et al (2001) found improvement in cardiac contractility of endurance trained subjects after consumption of Red Bull. The authors reported improved cardiac output which they attributed to a reduced end systolic volume.^5^ Umana-Alvarado et al (2004) reported lower rating of perceived exertion and also no improved run times in 11 male endurance runner when consuming 6 ml/kg ^-1^body weight of an energy drink 30 minutes before the two 10 km cross-country races.^6^ Byars et al (2006) reported the pre-exercise drink positively influenced energy and endurance on indices of maximal cardiorespiratory fitness.^7^

There are a limited number of studies supporting the use and qualitative ranking of these products. Several studies have been done among Iranian athletes.^8^,^9^ Furthermore, dietary intakes of Iranians have its own unique characteristics and it remains unknown whether consumption of these drinks would be of any benefit among athletes consuming Iranian dietary patterns or not.^10^ Therefore, the effectiveness of two caffeine and taurine-containing energy drinks (Red Bull produced in Austria and Hype produced in Netherlands) on VO_2max_, time to exhaustion, and heart rate variables and blood lactate levels in male athletes was evaluated. In addition, it was tested to see if the combined constituents of these drinks have ergogenic effects on exercise performance.

## Methods

### 

#### Subjects

After filling out questionnaires related to health and exercise history, from eligible athletes (healthy and active), ten male athlete students of Tehran University in the field of “physical education” (age: 22.4 ± 2.1 years, height: 180.8 ± 7.7 cm, weight: 74.2 ± 8.5 kg) were volunteered to take part in this study. The type of exercise they performed was regular aerobic and anaerobic exercises. Each male had given informed consent to participate in this research. Subjects included in this study if they were physically active and moderate caffeine users, and had no known sensitivity to test components or to any ingredient contained in the energy drinks. They also were not diabetic and take medication or nutritional supplementation and had no evidence of cardiac problems.^2^,^4^

#### Instruments

Subjects performed the exercise test on treadmill (Germany; GIGER); a Pulsimeter (Finnish; T1, 61-CODED, N2965, CE0537) estimated the heart rate (HR). Blood samples were collected from unpreferred hand mid-fingertips^11^ using a lactate analyzer (Analox P-LM55, UK). It should be noted that the analyzer had been calibrated with known lactate standards (5.0 and 15.0 mm). Stopwatch estimated the time to exhaustion. To determine VO_2max_, the standard formula was used.^12^

#### Exercise Test

The Bruce treadmill test was used as exercise test. The Bruce protocol is started at stage 1 with a speed of 2.74 km/h and grade of 10%. Every three minutes speed and grade were adjusted until the participants can no longer perform, ideally between 9 and 15 minutes. For example, stage 2 should be 4.02 km/h and 12% grade, stage 3 should be 5.47 km/h and 14% grade, and so on. This test was performed in three sessions and four days apart from each other.^2^

#### Procedures

One week prior to the first session of the test, subjects had a familiarization session to be instructed on how to perform each test.^2^,^13^ Also, before the testing, they were asked to wear comfortable, loose-fitting clothing.^7^ They performed the experiment the night before to avoid stimulants (e.g. alcohol, nicotine and other).^2^,^14^

Participants arrived at the laboratory of physical education and sport sciences faculty fasted at 8:00 A.M. and provided a standardized breakfast.^2^,^15^ The breakfast (a glass of boiled water, 45 grams bread and 1 gram butter) provided 1580 kJ (378 kcal) of energy and had 48% carbohydrate, 17% protein and 30% fat.^2^ After breakfast, the participants’ height (cm), weight (kg) and body composition, were measured by standard methods. In order to minimize the effect of diurnal variation, each test was arranged at a similar time of day (± 1 hour) with a room temperature of 22 ± 1°C and 63% relative humidity.^16^ Before the exercise test, basal blood lactate and also resting HR (by count of heartbeats within 60 seconds) were measured. Then, after five minutes of warm-up^17^ (measured by researcher’s assistant), a Bruce treadmill test in a controlled exercise physiology lab were administered. The test was then terminated and a cool-down period was initiated for several minutes. Immediately at the end of test, measurements of time to exhaustion and post-test HR were taken and for estimation of blood lactate, blood samples were collected two minute after exercise test.

#### Experimental Protocol

All participants performed three randomly assigned exercise experimental conditions: a) Red Bull energy drink; b) Hype energy drink; and c) placebo drink. In each session, in a randomized, placebo controlled, counterbalanced and double-blind design was consumed 6 ml/kg body weight^2^,^6^,^11^ Red Bull energy drink, Hype energy drink or placebo, 40 minutes before exercise test^5^,^11^ by athletes. The nutritional composition of two energy drinks is illustrated in table 1 and based on their body weight. The placebo beverage consisted of water with citrus substances.^7^,^11^

**Table 1:** Nutritional composition of two energy drinks (portion of 250 ml)

	Red Bull	Hype
Calories/portion (kcal)	122.5	99.1
Carbohydrates (g)	28.3	24.8
Protein (g)	0	< 1
Fat (g)	0	< 1
Caffeine (mg)	85	75
Taurine (mg)	1000	1000
Glucuronolacton (mg)	600	600
Other substances	B6, pantothenic acid, B12, water, niacin, inositol, sodium citrate, flavors, colors	B6, pantothenic acid, B12, water, niacin, inositol, sodium, flavors, colors

#### Statistical Analysis

Statistical significance was assessed using 3 × 3 (drinks vs. sessions) repeated measures analysis of variance (ANOVA). The least significant difference (LSD) test was performed for post-hoc analyses. The statistical package SPSS version 13 was used for statistical analysis. A value of p < 0.05 was considered statistically significant.

## Results

Descriptive statistics (Mean ± SD) for variables of three sessions in male athletes (n = 10) (Table 2) and percentage changes (Δ%) for variables of three sessions in male athletes (Table 3) were recorded.

**Table 2:** Descriptive statistics (Mean ± SD) for variables of three sessions in male athletes (n = 10)

	Placebo	Red Bull	Hype
VO_2max_ (ml.kg.min^-1^)	46.78 ± 4.12	52.86 ± 5.30	51.92 ± 3.90
Time to exhaustion (min)	13.02 ± 0.97	14.55 ± 1.37	14.41 ± 1.10
Pre-test heart rate (bpm)	74.20 ± 4.21	74.10 ± 3.35	74.50 ± 3.87
Post-test heart rate (bpm)	185.40 ± 4.81	187.80 ± 6.84	188.20 ± 4.08
Blood lactate 2 min before test (mmol.l)	1.19 ± 0.22	1.32 ± 0.31	1.27 ± 0.27
Blood lactate 2 min after the test (mmol.l)	13.95 ± 1.95	15.15 ± 3.26	14.63 ± 2.04

**Table 3:** Δ% for variables of three sessions in male athletes (n = 10)

	Red Bull-Placebo	Hype-Placebo	Red Bull-Hype
VO_2max_ (ml.kg.min^-1^)	11.5*	9.9*	1.8*
Time to exhaustion (min)	10.5*	9.7*	1%
Pre-test heart rate (bpm)	0%	0%	-1%
Post-test heart rate (bpm)	1%	1%	-0.2%
Blood lactate 2 min before the test (mmol.l)	0.1%	6%	4%
Blood lactate 2 min after the test (mmol.l)	0.8%	5%	3.4%

* Indicate significant difference

Using 3 × 3 repeated measures analysis of variance, significant differences were found in VO_2max_ (p = 0.001) and time to exhaustion (p = 0.000) values in three testing sessions. No significant difference was also found in pre-test HR (p = 0.592), post-test HR (p = 0.209), pretest blood lactate (p = 0.068) and post-test blood lactate (p = 0.069).

LSD test indicated a significant difference between VO_2max_in Red Bull and placebo (p = 0.01), Hype and placebo (p = 0.003) and no significant difference in Red Bull and Hype sessions (p = 0.576). Furthermore, a significant difference between time to exhaustion in Red Bull and placebo (p = 0.003), Hype and placebo (p = 0.001) and no significant difference in Red Bull and Hype sessions (p = 0.724) was observed.

## Discussion

The aim of this study was to investigate the effectiveness of Red Bull and Hype energy drinks on selected indices of maximal cardio-respiratory fitness and blood lactate levels in male athletes. Results of this study indicated that ingestion of Red Bull and Hype prior to exercise testing is effective on some indices of cardiorespiratory fitness but not on the blood lactate levels.

In the present study it was observed that Red Bull and Hype energy drinks caused an 11.5% and 9.9% increase in VO_2max_vs. placebo, respectively. Potentially, caffeine could have a number of actions that affect skeletal and heart muscle. It can inhibit adenosine receptors, increase sympathetic activity, and has direct intracellular action. The increase in VO_2max_may be attributed to ingredients such as caffeine, taurine, glucuronolactone and B group vitamins in these beverages. Caffeine has been shown to have desirable effects for performing athletes fighting fatigue, increasing energy, enhancing fatty acid metabolism and increasing skeletal muscle contractility. The stimulant effect of caffeine could make it effective for increasing alertness while performing in a fatigued state. The increased metabolism of free fatty acids has been proven to be useful during endurance exercise because of the glycogen sparing effects allowing an athlete to increase exercise time.^18^ Taurine can also aid in the contractile function of skeletal muscle. Taurine works to increase calcium content in the sarcoplasmic reticulum providing increased ability of the muscle to contract, amplifying muscle force generation.^1^,^19^ This may also reflect the action of glucuronolactone in providing additional energy resource.^4^ The Red Bull and Hype energy drinks contain also B group vitamins. Intake of B vitamins containing drinks or supplements can act to help replenish intermediates within the Kreb’s Cycle allowing for increased ATP production and potentially increase in energy supply to the working athlete, allowing for increased exercise time.^20^ The previous study of Baum et al (2001) supports this hypothesis suggesting that increases in VO_2max_might occur because the effects of caffeine and taurine may have on cardiac contractility.^5^

In this study it was found that Red Bull and Hype energy drinks caused up to 10.5% and 9.7% increase in time to exhaustion vs. placebo. The findings of Umana-Alvarado et al (2004) don’t support the present results.^6^ However, the present findings are in agreement with results of Geiß et al (1994).^3^ The increase in time to exhaustion may be due to absence of carbohydrate, caffeine and taurine. It is well established that carbohydrate is the preferred fuel for short-duration, high-intensity exercise and longer duration, higher intensity exercise^21^ and that depletion of muscle glycogen and blood glucose limit exercise duration and performance.^22^ Caffeine ingestion may enhance time to exhaustion because as mentioned earlier it has been implicated in increasing lipolysis from adipose tissue and thus enhancing fat oxidation resulting in spared stored glycogen during intense and prolonged exercise.^23^,^24^ Results of Zhang et al (2004) indicated significant increases in VO_2max_, time to exhaustion and maximal workload in exercise-test when consuming a taurine supplement. The authors postulate that this could be due to taurine attenuating exercise-induced DNA damage and increase of the capacity of exercise because of its cellular protective properties.^25^

In the present study, consumption of energy drinks did not affect our subjects HR. These findings support the results of Baum et al (2001), Bichler et al (2006) and Alford et al (2001).^4^,^5^,^26^ In contrast, lower HRs in endurance athletes after consuming of Red Bull energy drink has been reported.^3^ The lack of change in heart rate in the current study may be due to combination of taurine with caffeine in these beverages. No increase in HR when consuming caffeine suggest that taurine within the beverages may be doing something to alter cardiovascular physiology.^26^ Blood lactate levels changed after the current exercise test, but the change between groups were not significant. In the Bruce exercise intensity increases from one stage to another. Then, levels of blood lactate during intense exercise increased. No significant differences between groups indicated that these drinks do not affect blood lactate.

## Conclusions

In summary, it may be concluded that two caffeine and taurine-containing energy drinks have ergogenic effects on indices of maximal cardiorespiratory fitness in male athletes. Two energy drinks caused increase in VO_2max_, time to exhaustion, and no change in HR and blood lactate levels. The results of this research showed that the combined ingredients in these energy drinks may be responsible for the proposed effects.
